# Therapeutic potentials of allicin in cardiovascular disease: advances and future directions

**DOI:** 10.1186/s13020-024-00936-8

**Published:** 2024-07-02

**Authors:** Yijie Gao, Baofu Wang, Gaofeng Qin, Shichao Liang, Jiajie Yin, Hong Jiang, Mengru Liu, Xianlun Li

**Affiliations:** 1 National Integrated Traditional and Western Medicine Center for Cardiovascular Disease, China-Japan Friendship Hospital, Beijing, China; 2 Binzhou Medical University Hospital, Shandong, China; 3 Peking Union Medical College, Beijing, China; 4 Institute of Clinical Medical Sciences, China-Japan Friendship Hospital, Beijing, China

**Keywords:** Allicin, Diallyl thiosulfinate, Cardiovascular disease (CVD), Cardioprotective mechanisms, Nano drug delivery systems

## Abstract

Cardiovascular disease (CVD) remains the predominant cause of mortality and disability worldwide. Against this backdrop, finding effective drugs for the pharmacological treatment of CVD has become one of the most urgent and challenging issues in medical research. Garlic (*Allium sativum* L.) is one of the oldest plants and is world-renowned for its dietary and medicinal values. Allicin (*diallyl thiosulfinate*) is one of the primary natural active ingredients in garlic, which has been proven to have powerful cardioprotective effects and mediate various pathological processes related to CVD, such as inflammatory factor secretion, myocardial cell apoptosis, oxidative stress, and more. Therefore, allicin holds a promising application prospect in the treatment of CVD. This review summarized the biological functions of allicin and its potential mechanisms in CVD, including antioxidation, anti-inflammation, and anti-apoptosis effects. Reckoning with these, we delved into recent studies on allicin’s cardioprotective effects concerning various CVDs, such as atherosclerosis, hypertension, myocardial infarction, arrhythmia, cardiac hypertrophy, heart failure, and cardiotoxicity. Further, considering the tremendous advancement in nanomedicine, nanotechnology-based drug delivery systems show promise in addressing limitations of allicin’s clinical applications, including improving its solubility, stability, and bioavailability. Through this review, we hope to provide a reference for further research on allicin in cardioprotection and drug development.

**Graphical Abstract:**

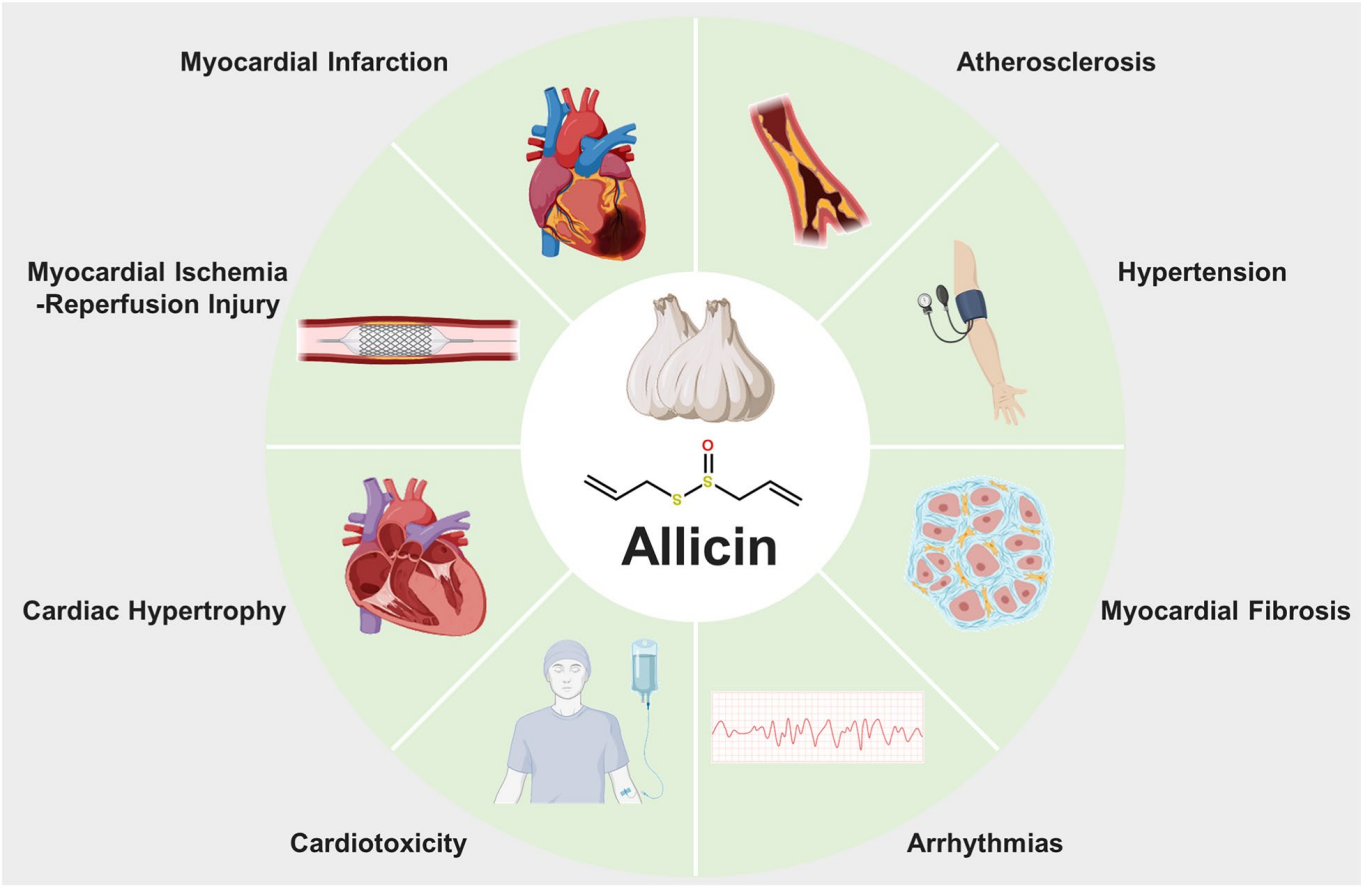

## Introduction

The term cardiovascular disease (CVD) encompasses a spectrum of conditions that inflict damage upon the heart and vascular system, encompassing hypertension, atherosclerosis, cardiomyopathy, myocardial infarction (MI), and heart failure (HF). These ailments stand as the primary cause of elevated morbidity and mortality on a global scale [1]. Despite the widespread utilization of cardioprotective pharmacotherapy, such as β-blockers, angiotensin-converting enzyme inhibitors (ACEIs), calcium channel blockers (CCBs), and statins, the prevalence of CVD continues to increase annually. Furthermore, chronic administration of these medications frequently results in a spectrum of adverse effects [2]. Therefore, the exploration and development of novel therapeutic agents for cardiovascular disease (CVD) is of paramount clinical significance. In this context, natural medicines are increasingly esteemed for their multiple targets, low cost, and low toxicity in the advancement of CVD treatments.

Garlic (*Allium sativum* L.) is widely utilized in cooking to enhance the flavor of dishes and has been globally employed since ancient times. Additionally, it possesses therapeutic properties and serves as a traditional medicinal plant used in various indigenous remedies worldwide [3]. The pharmacological benefits of garlic are extensive and well-documented, encompassing antioxidant, anti-inflammatory, antimicrobial, and anticancer properties. Recent epidemiological studies have revealed a negative correlation between garlic intake and the risk of cardiovascular events [4, 5], indicating the potential of garlic as a promising therapeutic agent for treating CVD. Modern pharmacological research has demonstrated that sulfur-containing compounds such as allicin, diallyl disulfide (DDS), diallyl trisulfide (DTS), and *S*-allyl-l-cysteine (SAC) constitute the main components of garlic [6].

Allicin, the most biologically active sulfur-containing compound of garlic, possesses various cardioprotective properties, including reducing blood pressure, regulating blood lipids, preventing atherosclerosis, and protecting against myocardial injury [7]. Therefore, allicin holds promising application prospects as a bioactive compound for treating CVD. This review presents a comprehensive overview of recent research on the cardioprotective mechanisms and potential clinical applications of allicin in CVD. Additionally, considering the rapid advancements in nanotechnology, novel delivery systems for allicin with improved stability, encapsulation efficiency, and bioavailability are also assessed, such as nanoparticles, liposomes, hydrogels, and nano‐emulsions. We hope to provide guidance and new ideas for drug development and clinical application of allicin in treating CVD.

## Allicin in the treatment of CVD

### The biological function of allicin

#### Chemistry properties of allicin

Allicin is present in white garlic and other Allium species such as field garlic (*A. vineale* L.), wild garlic (*A. ursinum* L.), elephant garlic (*A. ampeloprasum* L.), and alpine leek (*A. victorialis* L.), serves as the predominant biologically active sulfur-containing compound in garlic [6]. Allicin is the source of garlic’s distinctive strong, pungent odor and its spicy flavor profile. In its fresh state, garlic contains alliin (*S*-allyl-l-cysteine sulfoxide) but lacks free allicin. However, the mechanical crushing of garlic triggers alliinase to rapidly convert alliin into cytotoxic and odoriferous allicin [8].

Allicin, a colorless and oily liquid, exhibits limited solubility in water but is soluble in organic solvents, including ethanol, benzene, and ether. Allicin demonstrates low chemical stability and significant volatility, leading to its decomposition into smaller sulfur-containing compounds like 2-propene sulfenic acid, thioacrolein, and allyl alcohol at room temperature [9]. Furthermore, while allicin is unstable under heat and alkaline conditions, it demonstrates relative stability in acidic environments, particularly within a pH range of 5–7 [10]. In nature, garlic plants produce allicin upon tissue damage; however, it can also be synthesized chemically. For instance, a previous study successfully employed a binary system consisting of the plant enzyme alliinase and its substrate alliin to generate allicin [11]. Additionally, alliin serves as the precursor for allicin and can be obtained from garlic or synthesized through bromopropylation of cysteine followed by hydrogen peroxide oxidation [12].

#### Anti-inflammatory effects

After the occurrence of cardiovascular diseases, the body’s immune system can be activated, triggering an abnormal autoimmune response that mediates the onset of local inflammation and contributes to the progression of cardiovascular disease. Therefore, inflammation plays a significant role in the development and progression of CVD, including atherosclerosis, thrombosis, MI, and myocardial ischemia–reperfusion (MIR) injury [13]. Targeting anti-inflammatory therapies has emerged as a promising strategy. Accumulating evidence indicates that increased secretion and circulation of inflammatory markers such as Interleukin-6 (IL-6), tumour necrosis factor-alpha (TNF-α), and Interleukin-1β (IL-1β) are associated with the development and exacerbation of CVD [14]. Jessica et al. [15] discovered that allicin can attenuate both transcript and protein expression of pro-inflammatory cytokines IL-1β, IL-6, and IL-12β, while simultaneously enhancing the expression of the anti-inflammatory cytokine IL-10 in lipopolysaccharide (LPS)-induced macrophage model. The administration of allicin exhibited a significant reduction in serum levels of pro-inflammatory cytokines IL-1β, IL-6, and TNF-α across multiple animal models [16, 17].

Recent research indicated that Toll-like receptor 4 (TLR4) and the nuclear factor kappa B (NF-κB) signalling pathway play a role in promoting the activation of cytokines such as TNF-α, IL-6, and IL-1β to exacerbate inflammation, leading to subsequent myocardial tissue damage [18]. Inhibition or absence of TLR4 or NF-κB, which are critical regulators in pro-inflammatory cascades, had been shown to reduce left ventricular remodelling extent and improve cardiac function [19]. Ca^2+^/calmodulin-dependent protein kinase II (CaMKII) is an essential protein involved in regulating intracellular calcium transport processes and cardiomyocyte contractile function. It mainly phosphorylates calcium transport-related proteins, which increases calcium influx and strengthens cardiomyocyte contractility, thereby affecting cardiac function [20]. Recent findings suggested that CaMKII can trigger NF-κB-mediated pro-inflammatory gene expression and the inflammasome activation via TLR4 to promote the release of downstream pro-inflammatory factors in response to pressure overload [21]. In addition, the NOD-like receptor pyrin domain-containing protein 3 (NLRP3) inflammasome, a multiprotein signalling complex, is also assembled and activated in response to damage-associated molecular patterns (DAMPs), which then catalyzes active forms of pro-inflammatory cytokines, such as IL-1β and IL-18. Therefore, the activation of the NLRP3 inflammasome is considered to be a key link in aggravating vascular and cardiac inflammation [22]. Takeshiet et al. [23] found that allicin significantly reduced serum IL-1β, IL-6, and TNF-α levels, improved calcium homeostasis in cardiomyocytes, downregulated calcium transportation-related CaMKII and inflammation-related NF-κB and NLRP3 expression, which was observed in smooth muscle cells and cardiomyocytes. Additional experiments demonstrated that allicin abrogated inflammation and myocardial fibrosis by blocking the activation of NF-κB and Smad 2/3 signalling, respectively [24]. Moreover, allicin attenuated the LPS-induced increase in NLRP3, pro-IL-1β, and IL-1β proteins. Silencing of Nrf2 by siRNA (siNrf2) significantly attenuated the allicin-induced increase in cell viability and HO-1 and decrease in NLRP3 protein in LPS-stimulated H9C2 cells [25].

#### Anti-oxidative stress

Oxidative stress plays a pivotal role in the pathophysiological mechanisms of various cardiovascular diseases and is involved in all stages of CVD onset and progression. Its mechanism encompasses complex signalling molecules and regulatory pathways, thereby emphasizing the pivotal role of antioxidant stress in the treatment of CVD. Oxidative stress refers to an imbalance between reactive oxygen species (ROS) generation and antioxidants within the body or cells when exposed to diverse detrimental stimuli, ultimately resulting in tissue damage [26]. ROS are the main compounds involved in oxidative stress reactions, including oxygen free radicals such as superoxide, hydroxyl radicals, singlet oxygen, and peroxyl radicals, as well as non-radicals that can generate free radicals, such as hydrogen peroxide (H_2_O_2_) and hypochlorous acid [27]. A study discovered that pre-incubation with allicin (0.3–10 μM) could concentration-dependently mitigate the increase of intracellular ROS induced by H_2_O_2_ in H9C2 cells and protect these cells against H_2_O_2_-induced damage [28]. Wang et al. [29] demonstrated that allicin exhibited a protective effect against injury induced by high glucose/hypoxia in aortic endothelial cells by significantly reducing ROS production, which may involve inhibiting the PKC pathway and regulating the HIF-1α signalling pathway.

Malondialdehyde (MDA) is the final product of lipid peroxidation reactions caused by oxygen free radicals, displaying significant cytotoxicity and exacerbating cell membrane damage. It is commonly used as an indicator reflecting the degree of oxidative stress and the amount of oxygen-free radical generation [30]. Various endogenous enzymes and non-enzyme antioxidants present in normal myocardium are sufficient to counteract the cytotoxic products of ROS, including antioxidant enzymes such as superoxide dismutase (SOD), glutathione peroxidase (GSH-Px), catalase (CAT), and peroxidase (Prx). Allicin supplementation can significantly reduce serum MDA levels in rabbits fed with a 1% high-cholesterol diet, while simultaneously increasing serum HDL-C, GSH, and SOD levels [31]. Additionally, allicin can enhance the activity of SOD, CAT, and GSH-Px, thereby improving both systolic and diastolic function in the myocardium of rats experiencing MIR injury [16]. This mechanism was partly associated with alleviating the activation of the p38 signalling pathway. Liu et al. [24] indicated that allicin protected cardiac function and prevented the development of cardiac hypertrophy through ROS-dependent mechanisms involving multiple intracellular signalling, such as ERK1/2, JNK1/2, and AKT signalling pathways.

#### Anti-apoptosis

Apoptosis is a programmed cell death process that is activated by various signalling cascades and regulated by intricate networks. Two extrinsic and intrinsic signalling pathways mainly regulate apoptosis. The extrinsic apoptosis pathway is initiated by death ligands triggering death receptor signalling on the cell surface. The intrinsic apoptosis pathway is activated by oxidative stress, calcium overload, DNA damage, and activation of various transcription factors within the mitochondria, leading to an intracellular apoptosis pathway. Regardless of whether it follows the extrinsic or intrinsic pathway, apoptosis ultimately leads to the activation of effector caspase-3 and induces cell death. In CVD, apoptosis of myocardial cells can exacerbate pathological processes by reducing their numbers. Therefore, it is crucial to reduce and inhibit myocardial cell apoptosis for effective treatment of CVD. A study demonstrated that allicin treatment effectively modulated apoptosis-related proteins, leading to a reduction in the expression levels of Bax and cleaved caspase-3, while simultaneously increasing the expression levels of Bcl-2 and cytosolic cytochrome C [32]. Deng et al. [33] found that allicin treatment enhanced cell viability and reduced apoptosis from 13.5 ± 1.2% to 6.11 ± 0.15% compared with hypoxia/reoxygenation in H9C2 cells.

**Fig. 1:**
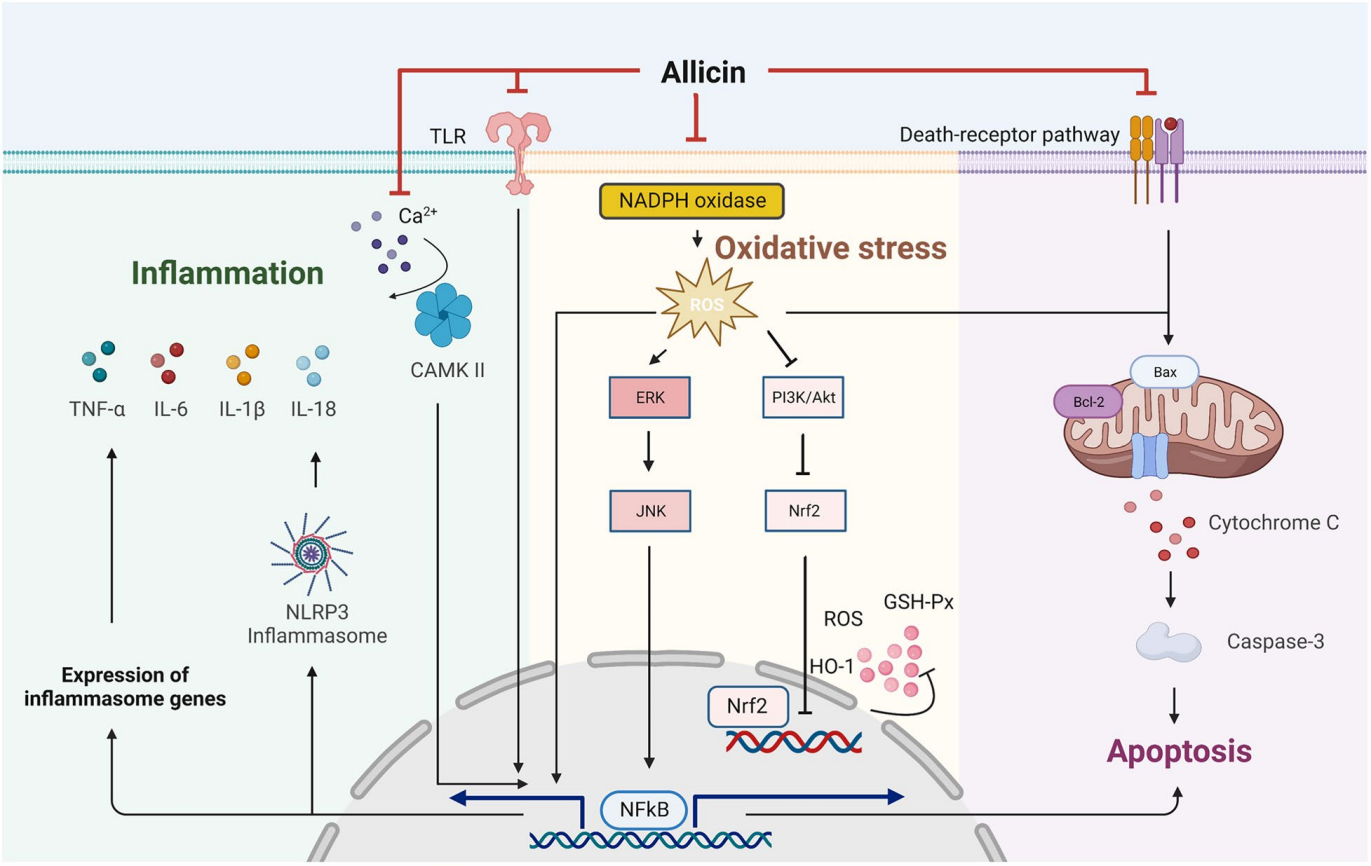
Allicin can exert cardioprotective effects on CVD through various pathways, including anti-apoptosis, antioxidant stress reduction, and anti-inflammatory effects. Allicin regulates the expression of caspase-3 by increasing the level of Bcl-2/Bax, thereby inhibiting apoptosis triggered by the death-receptor pathway. Allicin exhibits inhibitory effects on multiple pathways involved in ROS-mediated oxidative stress, such as the Nrf2 pathway, JNK pathway, and NF-ĸB pathway. Furthermore, allicin can inhibit the activation of inflammatory response by blocking both TLR/NF-ĸB pathway and Ca^2+^-mediated CaMKII signaling pathway. *BAX* BCL2-associated X protein, *Bcl-2* B-cell lymphoma 2, *CaMKII* Ca^2+^/calmodulin-dependent kinase II, *JNK* c-Jun N-terminal kinase, *NF-ĸB* nuclear factor-kappaB, *Nrf2* nuclear factor erythroid-2-related factor 2, *ROS* reactive oxygen species, *TLR* Toll-like receptor 4

Guo et al. discovered that allicin inhibited the high glucose-induced activation of caspase-3 and nuclear translocation of NF-κB, which appeared to suppress elevated glucose-induced cardiomyocyte apoptosis by inhibiting NADPH oxidase-related ROS and its downstream JNK/NF-κB signalling pathway [34]. Ma et al. [35] established an MI model by ligating the left coronary artery, demonstrating that the myocardial apoptotic index was also markedly lowered in allicin-treated rats. Furthermore, allicin can effectively reduce cardiomyocyte apoptosis in rat models of MI by significantly regulating the JNK signalling pathway [36]. Mohamed et al. [37] observed that methotrexate (MTX), a chemotherapy drug known for its severe cardiac side effects, induced alterations in the cardiac histopathology and enhanced the caspase-3 expression in the rat cardiac tissues, indicating the exacerbation of cardiomyocyte apoptosis. Allicin administration at a dosage of 20 mg/kg orally for 2 weeks effectively attenuated MTX-induced apoptosis of myocardial cells. Similarly, intraperitoneal injection of doxorubicin (DOX) resulted in significant activation of caspase-3 in myocardial tissue, which was markedly alleviated by pretreatment with allicin and restored cardiac structure [17] (Fig. 1).

### Atherosclerosis

Atherosclerosis (AS), a chronic arterial disease characterized by arterial inflammation and lipid deposition within the vessel wall intima, is closely associated with the development of CVD [38]. Elevated levels of triglycerides (TG), low-density lipoprotein cholesterol (LDL-C), and total cholesterol (TC) have been identified as significant contributors to AS. In individuals with hypercholesterolemia, a daily intake of 9.6 mg of allicin resulted in a reduction of TC by 4.2% and LDL-C by 6.6% [39]. Similarly, Lu et al. [40] discovered that allicin decreased TC, TG, and LDL-C levels in mice fed a high-cholesterol diet while also mitigating oxidative stress damage, inflammatory responses, vascular dysfunction, and aortic lesions. Lin et al. [41] found that allicin reduced cholesterol levels in foam cells and enhanced cholesterol efflux by activating the peroxisome proliferator-activated receptor γ (PPARγ)/liver X receptor alpha (LXRα) signalling pathway, thereby reducing lipid accumulation. Nantiya et al. [42] demonstrated that allicin possessed hypolipidemic effects by upregulating low-density lipoprotein receptor (LDLR) expression through the activation of sterol regulatory element binding proteins 2 (SREBP2) and downregulating proprotein convertase subtilisin/kexin type 9 (PCSK9) expression via the suppression of hepatocyte nuclear factor 1α (HNF1α), thereby enhancing the uptake of LDL by HepG2 cells. Moreover, as mentioned earlier, the study also revealed that the efficacy of allicin (200 μM) in lowering blood lipids was comparable to atorvastatin’s effect (10 μM).

Additionally, allicin has been shown to effectively mitigate the risk factors associated with atherosclerosis (AS) and delay its onset and progression. Homocysteine (Hcy) is an independent risk factor for AS. Liu et al. [43] found that allicin effectively reduced the carotid intima-media thickness (cIMT), TC, and TG by lowering plasma Hcy levels, thereby preventing the development of AS in patients with hyperhomocysteinemia. In recent years, an increasing body of research indicated that alterations in the composition of gut microbiota and the metabolites derived from it have significant implications for the progression of AS [44]. The gut microbiota-generated metabolite, trimethylamine N-oxide (TMAO), is widely recognized as a significant risk factor in promoting the progression of atherosclerosis [45]. Elevated blood levels of trimethylamine oxide (TMAO) have been demonstrated to be associated with an increased risk of major adverse cardiovascular events and all-cause mortality [46]. TMAO is implicated in inflammation and oxidative stress, cholesterol metabolism, bile acid metabolism, and foam cell formation in AS [47]. A recent study found that the consumption of raw garlic juice for 1 week inhibited TMAO formation and increased the relative abundance of beneficial gut bacteria in individuals with high TMAO levels [48]. Allicin also significantly decreased serum TMAO levels and attenuated aortic lesions in carnitine-induced apolipoprotein E-deficient (ApoE) mice. These findings indicate that allicin may play a vital role in delaying the occurrence and progression of AS through multiple targets and mechanisms (Table 1).

**Table 1:** The Mechanisms of allicin in atherosclerosis

Research model	Model establishment	Intervention methods	Drug effects	Main molecular mechanisms	Citation
Mild to moderate hypercholesterolemic patients	–	4.8 mg twice daily for 12 weeks	Reducing lipid	⬇ TC, LDL-C	[39]
Hypercholesterolemic male ICR mice	Atherogenic high-cholesterol diet	5, 10, or 20 mg/kg, oral daily, 12 weeks	Lowering lipid; reducing the lipid accumulation in hepatic cells	⬇ TC, TG, LDL-C, GLU	[40]
THP-1 macrophage-derived foam cells	50 mg/ml ox-LDL for 48 h	5 g/l for 24 h	Promoting cholesterol efflux, reducing lipid accumulation	⬆ ABCA1, PPARγ, LXRα	[41]
HepG2	–	100, 200 μM for 24 h	Hypolipidemic	⬇ PCSK9, HNF-1α ⬆ LDLR, SREBP2, LDL uptake	[42]
CAD patients with HHcy	–	40 mg thrice daily for 12 weeks	Decreasing carotid artery IMT, reducing lipid	⬇ Hcy, TC, TG	[43]
l-Carnitine-fed C57BL/6 mice	l-Carnitine-fed	10 mg/kg in 0.5% CMC per day for 2 weeks	Regulating gut bacteria, reducing aortic plaques	⬇ serum TAM, TMAO and γBB (C57BL/6J) ⬇ TMAO_MAX_ and TMAO_AUC_ in plasma and urine (subjects) ⬇ serum d9-TMA and d9-TMAO (ApoE^−/−^) ⬆ enriching certain beneficial and anti-inflammatory gut commensal bacteria (subjects)	[48]
ApoE^−/−^ female mice	l-Carnitine-fed	10 mg/kg in 0.5% CMC per day for 15 weeks			
High-TMAO subjects	–	55 ml of raw garlic juice (48 mg of allicin equivalent) once a day during dinner for 1 week			

*ABCA1* ATP binding cassette transporter A1, *CAD* coronary artery disease, *CMC* carboxymethyl cellulose, *Hcy* homocysteine, *HDL* high-density lipoprotein cholesterol, *HHcy* hyperhomocysteinemia, *HNF-1α* hepatocyte nuclear factor-1 alpha, *IMT* intima‑media thickness, *GLU* glucose, *LDH* lactate dehydrogenase, *LDL* low-density lipoprotein, *LDL-C* low-density lipoprotein cholesterol, *LXRα* liver X receptor alpha, *TC* total cholesterol, *TG* triglyceride, *TMA* trimethylamine, *TMAO* trimethylamine *N*-oxide, *PPARγ* peroxisome proliferator-activated receptor γ, *PCSK9* proprotein convertase subtilisin/kexin type 9, *SREBP2* sterol regulatory element binding proteins 2

### Hypertension

Hypertension is a significant risk factor for CVD. The pathogenesis of hypertension is commonly believed to involve endothelial dysfunction and increased peripheral vascular resistance [49]. Elkayam et al. [50] discovered that the administration of 80 mg/kg/day of allicin for 6 weeks resulted in reduced systolic blood pressure in spontaneously hypertensive rats (SHRs). The impact of allicin on blood pressure and renal function was comparable to that of losartan in rats with chronic kidney disease, and the antihypertensive effect of allicin was associated with the upregulation of angiotensin II receptor type 2 (AT2R) and endothelial nitric oxide synthase (eNOS) as vasodilators [51]. On the other hand, studies found that the anti-hypertensive effect of allicin can be primarily attributed to its easy degradation into organosulfur compounds in the presence of thiols, becoming an effective endogenous hydrogen sulfide (H_2_S) donor in the body [52]. H_2_S has been established as a potent gaseous signalling molecule with vasodilatory activity, playing a crucial role in maintaining basal blood pressure and contributing to hypertension development [53]. Cui et al. [54] demonstrated that allicin promotes vasorelaxation through both the NO-soluble guanylate cyclase-cyclic guanosine monophosphate (NO-sGC-cGMP) pathway and prostacyclin-adenylyl cyclase-cyclic adenosine monophosphate (PGI2-AC-cAMP) pathway via H_2_S production, thus exhibiting robust anti-hypertensive effects in SHRs. Additionally, remodelling caused by hypertension is considered the pathological basis of target organ damage [55]. Liu et al. [23] concluded that allicin effectively ameliorated vascular and cardiac remodelling induced by hypertension through inhibition of the CaMKII/NF-κB signalling pathway, reduction in serum levels of IL-1β, IL-6, and TNF-α, improvement in calcium homeostasis in cardiomyocytes, and downregulation of the NLRP3 inflammasome. In summary, these studies provide evidence for the beneficial antihypertensive effects of allicin (Table 2).

**Table 2:** The mechanisms of allicin in hypertension

Research Model	Model establishment	Intervention methods	Drug effects	Main molecular mechanisms	Citation
Male spontaneously hypertensive rats	Spontaneously hypertension	14 mg/kg, intragastric administration for 4 weeks	Anti-hypertensive, anti-cardiac remodeling	⬇ SBP, DBP, PCNA, α-SMA, LVWT, cardiac fibro collagen, CaMK II, NF-κB p65, NF-κB p50, NLRP3, IL-6, TNF-α, IL-1β ⬆ SM22α, cardiomyocytes contraction amplitude and Ca^2+^ transient amplitude	[23]
Male spontaneously hypertensive rats	Spontaneously hypertension	80 mg/kg/day, powder mixed with ground chow for 6 weeks	Regulating BP, reducing lipid	⬇ SBP, TG	[50]
Male Wistar rats with CKD	Renal ablation (5/6 nephrectomy)	40 mg/kg/day, oral for 3 and 6 weeks	Anti-hypertensive, antioxidant, nephroprotective	⬇ SBP, urinary excretion of NAG, albuminuria, nephrin, fibrosis in the renal cortex, oxidized proteins and lipid peroxidation in the cortex and medulla of the kidney, HO-1 ⬆ Glomerular and tubular area, AT1R, AT2R, Nrf2, keap1, CAT, SOD, eNOS	[51]
Male spontaneously hypertensive rats	Spontaneously hypertension	7 and 14 mg/kg, intragastric administration for 4 weeks	Anti-hypertensive	⬇ SBP, DBP ⬆ EDHF, NO-sGC-cGMP, PGI_2_-AC-cAMP, H_2_S	[54]

*α-SMA* alpha-smooth muscle actin, *AT1R* angiotensin II type 1 receptor, *AT2R* angiotensin II type 2 receptor, *BP* blood pressure, *CaMKII* Ca^2+^/calmodulin-dependent kinase II, *CAT* catalase, *cAMP* cyclic adenosine monophosphate, *cGMP* cyclic guanosine monophosphate, *CKD* chronic kidney disease, *DBP* diastolic blood pressure, *eNOS* endothelial nitric oxide synthase, *EDHF* endothelium-derived hyperpolarizing factor, *HO-1* heme oxygenase-1, *H*_*2*_*S* hydrogen sulfide, *Keap1* Kelch-like ECH-associated protein1, *IL-1β* Interleukin-1β, *IL-6* Interleukin-6, *LVWT* left ventricular wall thickness, *NAG* *N*-acetyl-d-glucosaminidase, *NLRP3* NOD-like receptor pyrin domain-containing protein 3, *Nrf2* nuclear factor erythroid-2-related factor 2, *NO-sGC-cGMP* nitric oxide-soluble guanylate cyclase-cyclic guanosine monophosphate, *NF-κB* nuclear factor-kappa B, *PCNA* proliferating cell nuclear antigen, *PGI2* prostaglandin, *SBP* systolic blood pressure, *SOD* superoxide dismutase, *TNF-α* tumor necrosis factor-alpha

### Myocardial infarction and myocardial ischemia–reperfusion injury

Cardiomyocyte apoptosis is a crucial pathological factor associated with MI. The extent of cardiomyocyte apoptosis positively correlates with the severity of MI [56]. Allicin had been reported to reduce infarct size and improve cardiac function by inhibiting the Bcl-2/Bax signaling pathway-dependent cell apoptosis [35]. Furthermore, the administration of Allicin can dose-dependently decrease levels of creatine kinase and lactate dehydrogenase after MI. In a rat model of MI induced by subcutaneous injection of isoproterenol, allicin significantly alleviated cardiomyocyte apoptosis by regulating the c-Jun N-terminal kinase (JNK) signalling pathway. Its inhibitory effect may be attributed to the activation of the endogenous protective eNOS/NO-mediated antioxidant stress signalling pathway [36]. In addition, the H_2_S synthesizing enzymes cystathionine-β-synthase (CBS) and cystathionine-γ-lyase (CSE) in myocardial tissues of rats decreased after MI, leading to a significant reduction in H_2_S in myocardial tissues and blood. As an influential donor of H_2_S within the body, Allicin can elevate CBS and CSE levels in myocardial tissues, maintain Ca^2+^ homeostasis of cardiomyocytes, regulate coronary artery vasodilation, and thus exert protective effects on ischemic myocardium [57].

Myocardial ischemia–reperfusion (MIR) injury is a complicated pathophysiological process characterized by intense inflammatory reactions, cardiomyocyte apoptosis, oxidative stress, and Ca^2+^ overload. Allicin significantly attenuated the release of inflammatory factors such as IL-6, TNF-α, and IL-8 in the serum after MIR by inhibiting the activation of the p38/MAPK signalling pathway. Additionally, allicin can enhance the activities of SOD, CAT, and GSH-Px to ameliorate myocardial contractility and relaxation function [16]. Deng et al. [33] simulated MIR injury in vitro using a hypoxia-reoxygenation (HR) model with pig cardiomyocytes and observed that allicin significantly reduced the expressions of IL-6 and TNF-α after HR, as well as mitigating the loss of mitochondrial membrane potential.

This study also revealed that allicin decreased the apoptosis rate of HR-induced cardiomyocytes, reduced the expressions of apoptosis-related proteins Bax, cleaved caspase-3, and cytosolic cytochrome C, and increased the expression of Bcl-2. Gao et al. [58] pointed out that pretreatment with allicin reduced infarct size and improved cardiac function in MIR mice partially by inhibiting the PI3K-mediated GRK2/PLC-γ/IP3R signalling pathway, thereby suppressing Ca^2+^ overload-induced cardiomyocyte apoptosis. These findings suggest that allicin confers protection to cardiomyocytes against HR damage through attenuation of apoptosis, inflammation, and mitochondrial injury. Additionally, Liu et al. [59] discovered that allicin promoted angiogenesis and safeguards myocardial tissue from HR injury by modulating miR-19a-3p to inhibit the PI3K/AKT signalling pathway (Table 3).

**Table 3:** The mechanisms of allicin in myocardial infarction and ischemia–reperfusion injury

Research Model	Model establishment	Intervention methods	Drug effects	Main molecular mechanisms	Citation
Male SD rats with MIR	Ligating LAD to induce ischemia for 30 min, followed by 4 h of reperfusion	50 mg/kg intraperitoneal injection at 0.5 h before the induction of ischemia	Cardioprotection, suppressing inflammation, suppressing oxidative stress	⬇ cTnI, CK-MB, LVEDP, MDA, TNF-α, IL-6, IL-8, p-p38, p38 ⬆ LVSP, SOD, CAT, GPx	[16]
HR primary porcine cardiomyocytes	Hypoxia 2 h, reoxygenation 3 h	20 μg/ml for 4 h (validation concentration)	Reducing apoptosis, anti-inflammation, reducing mitochondrial injury	⬇ Bax, cleaved caspase-3, Cyt-c, TNF-α, IL-6, ROS, HIF-1α, ET-1, TGF-β ⬆ Bcl-2, eNOS, PGC1-α	[33]
Wistar rats with MI	Ligating LAD	1.2, 1.8, and 3.6 mg/kg, intraperitoneally daily for 3 weeks	Cardioprotection, improving cardiac function	⬇Infarct area, CK, LDH, myocardial apoptosis, LVID, Bax ⬆ LVAWd/s, EF, FS, SV, Bcl-2	[35]
Male SD rats with myocardial ischemia	Injection of ISO (85 mg/kg/day) for 2 consecutive days	1.2, 1.8, and 3.6 mg/kg, intraperitoneally daily for 1 week	Reducing oxidative stress damage, reducing cardiomyocyte apoptosis	⬇ Myocardial fibrosis, inflammatory cell infiltration, MDA, p-iNOS, iNOS, Cyt-c, caspase-3, caspase-9 ⬆ SOD, CAT, GSH-Px, eNOS, Bcl-2, Bax, JNK, p-JNK	[36]
Male SD rats with MI	Ligating LAD	7 and 14 mg/kg intraperitoneal injection for 1 week	Cardioprotection, improving cardiac function, regulating coronary artery vasodilation	⬇ cTnT, LDH, myocardial infarction area, LVID, Ca^2+^ release by the ryanodine receptor, SR Ca^2+^ leakage ⬆ H_2_S in serum and myocardial tissue, LVAWd/s, EF, FS, SV, CSE, CBS, opening of K_ATP_, Ca^2+^ transients amplitude, Ca^2+^ uptake via SERCA, Ca^2+^ removal via the NCX, myofilament sensitivity, SR Ca^2+^content	[57]
Male Wistar rats with MIR	Ligating LAD to induce ischemia for 30 min, followed by 3 h of reperfusion	1.88 mg/kg, orally via gavage, 12 h before inducing ischemia	Anti-apoptosis, improving cardiac function	⬇ LVIDd/s, infarct size, Bax, cleaved caspase-3, cleaved caspase-9, GRK2, p-GRK2, p-CaMKII, p-PLC-γ, p-IP3R ⬆ LVAWd/s, LVEF, Bcl-2, PI3K, p-PI3K	[58]
Male C57BL6 mice with MIR	Ligating LAD to induce ischemia for 30 min, followed by 3 h of reperfusion	3 mg/ml 10 min + 5 mg/ml 15 min (intravenously) before MIR	Promoting angiogenesis, improving cardiac function	⬇ LVID, cardiac fibrosis areas, miR-19a-3p ⬆ EF, FS, LVAWd/s, neovascularization numbers, LDH, COX-2, p-AKT, p-PI3K, p-mTOR, VEGF, MMP-2	[59]

*CaMKII* Ca^2+^/Calmodulin-dependent protein kinase II, *CBS* cystathionine beta-synthase, *CK* creatine kinase, *COX-2* cyclooxygenase-2, *CSE* cystathionine-γ-lyase, *cTnT* cardiac troponin T, *Cyt-c* cytosolic cytochrome C, *EF* ejection fraction, *ET-1* endothelin-1, *FS* short-axis shortening, *GPx* glutathione peroxidase, *JNK* c-Jun N-terminal kinase, *LVAWd/s* left ventricular anterior wall diastolic and systolic thicknesses, *MDA* malondialdehyde, *MIR* myocardial ischemia reperfusion, *MMP-2* matrix metalloproteinas-2, *NCX* Na^+^/Ca^2+^ exchanger, *p-mTOR* p-mammalian target of rapamycin, *PLC-γ* recombinant phospholipase C gamma, *p-PI3K* *p*-phosphatidylinositol-3-kinase, *iNOS* inducible nitric oxide synthase, *SV* stroke volume, *SD* Sprague Dawley, *SERCA* sarco/endoplasmic reticulum Ca^2+^-ATPase, *SR* sarcoplasmic reticulum, *TGF-β* transforming growth factor-β, *VLDL* very low-density lipoprotein cholesterol, *VEGF* vascular endothelial growth factor

### Cardiac hypertrophy

Cardiac hypertrophy (CH) is an adaptive compensatory response of the heart to oxidative stress, hemodynamic load, angiotensin II (Ang II), hormones, and other stimulating factors [60]. Persistent CH can lead to cardiac dilation, heart failure, and even sudden cardiac death. Pressure overload induces CH accompanied by an elevation in ROS, which activates various hypertrophy signals and factors. However, treatment with a free radical scavenger effectively mitigates the hypertrophic response [61]. Li et al. [62] discovered that allicin activates the Nrf2/HO-1 antioxidant signalling pathway to decrease ROS levels, carbonylated proteins, and thiobarbituric acid reactive substances (TBARS), while enhancing GPx activity. This prevented the development of cardiac remodelling and retards the progression of CH in Ang II-induced CH rats. Liu et al. [24] found that allicin markedly inhibited hypertrophy responses induced by Ang II or pressure overload and increased ROS generation and NADPH oxidase activity in an animal model of CH. The underlying mechanism might be the ROS-dependent ERK1/2, JNK1/2, and AKT signalling pathways blockage.

A recent study revealed that allicin significantly improved cardiac function in rats with abdominal aortic constriction (AAC)-induced CH [63]. Specifically, allicin not only reduced CH marker proteins such as brain natriuretic peptide (BNP) and β-myosin heavy chain (β-MHC) but also decreased autophagy marker proteins including Beclin-1 and LC3-II in the hearts of AAC-induced rats and Ang II-treated neonatal rat cardiomyocytes. The beneficial effects of allicin were found to be antagonized by the pharmacological inhibitor of the mammalian target of rapamycin (mTOR). These findings elucidate a potential mechanism through which allicin attenuates CH by inhibiting excessive autophagy via activation of PI3K/Akt/mTOR and MAPK/ERK/mTOR signaling pathways. Interestingly, considering the significant correlation between cardiac microvascular damage and the development of CH, Shi et al. [64] discovered that allicin improved the distribution and expression of platelet endothelial cell adhesion molecule-1 (PECAM-1) while activating the PECAM-1-PI3K-Akt-eNOS signaling pathway. This activation facilitated migration and angiogenesis in cardiac microvascular endothelial cells, thereby advancing the modification ability of their shear adaptation. These findings provide potent support for the potential therapeutic application of allicin in managing CH (Table 4).

**Table 4:** The mechanisms of allicin in cardiac hypertrophy

Research Model	Model establishment	Intervention methods	Drug effects	Main molecular mechanisms	Citation
Primary cardiac myocytes	1 μM AngII for 48 h	Pretreated for 60 min before interfering with AngII	Anti-inflammation, reducing cardiac hypertrophy, improving cardiac function	⬇ LVESD, LVEDD, LVPWd, ANP, BNP, Myh7, ROS, IL-6, TNF-α, MCP-1, p-ERK1/2, ERK1/2, p- JNK1/2, JNK1/2, p-Akt, Akt, p-p38/p38, p-p85/p85, p-GSK3β, GSK3β, NF-κB, p-Smad2/3, Smad2/3, CTGF ⬆ FS	[24]
Cardiac fibroblast					
Male C57/B6 mice with pathological cardiac hypertrophy	Aortic banding	50 mg/kg per day for 1 week before aortic banding surgery and 8 weeks after surgery			
Male SD rats with cardiac hypertrophy	AngII (250 ng/kg per min) through mini-osmotic pumps implanted subcutaneously for 2 weeks	180 mg/kg/day contained in the diet at the 4 weeks after the surgery for 8 consecutive weeks	Alleviating cardiac remodeling, reducing cardiac hypertrophy, improving cardiac function	⬇ SBP, DBP, LV mass, posterior wall thickness, LVEDd, IVSd, LVPWd, collagen and collagen I/III mRNA expression, ROS ⬆ EF, FS, GPx, GSH, GPx activities, levels of GSH and T-AOC, protein levels and mRNA expression of Nrf2, NQO1, and γ-GCS, HO-1	[62]
Male Wistar rats with pathological cardiac hypertrophy	Abdominal aortic constriction	5, 10, and 2 mg/kg, intraperitoneal injection for 4 consecutive weeks	Inhibiting excessive autophagy, improving cardiac function	⬇ LVEDP, HW/BW, LVW/BW, HW/TL, BNP, β-MHC, LC3-II, Beclin-1 ⬆ LVSP, p-Akt, p-PI3K, p-ERK	[63]
Primary neonatal rat cardiac myocytes	AngII 100 nM for 48 h	25, 50 and 100 μM for 48 h	Improving the function of CMECs, improving cardiac function	⬇ LVPWd, LVIDd/s, BNP, β-MHC, caspase-3, RIP3 ⬆FS, EF, cardiac microvascular density, p-eNOS/eNOS, PECAM-1, Ang-2, PDGFR-β, NO, p-PI3K, PI3K, p-Akt, Akt	[64]
Male Wistar rats with pathological cardiac hypertrophy	Abdominal aortic constriction	5, 10, and 2 mg/kg, intraperitoneal injection for 4 consecutive weeks			
CMECs	Ang II 100 nM for 24 h	25, 50, and 100 μM for 24 h			

*AngII* angiotensin II, *Ang-2* angiopoietin-2, *BNP* b-type natriuretic peptide, *β-MHC* β-myosin heavy chain, *CMECs* cardiac microvascular endothelial cells, *CTGF* connective tissue growth factor, *ERK1/2* extracellular signal-regulated kinase, *GSK3β* glycogen synthase kinase 3 beta, *HW/BW* heart weight to body weight ratio, *LVEDP* left ventricular end-diastolic pressure, *LVESD* left ventricular end-systolic internal diameter, *LVPWd* left ventricular posterior wall diastolic thicknesses, *LVSP* left ventricular systolic pressure, *LVW/BW* left ventricle weight to body weight ratio, *HW/TL* heart weight to tibia length ratio, *MCP-1* monocyte chemoattractant protein-1, *Myh7* myosin heavy chain 7, *Nrf2* nuclear factor erythroid-2-related factor 2, *PDGFR-β* platelet-derived growth factor receptor-β, *PECAM-1* platelet endothelial cell adhesion molecule-1, *p-GSK3β* *p*-glycogen synthase kinase 3 beta, *RIP3* receptor interacting protein 3, *T-AOC* total antioxidant capability, *γ-GCS* γ-glutamylcysteine synthetase

### Myocardial fibrosis

Myocardial fibrosis is the result of excessive fibrillar collagen synthesis and deposition, which determines the clinical course and outcome of HF patients [65]. The transforming growth factor-β (TGF-β)/Smad signalling pathway has been proven to play a crucial role in the progression of myocardial fibrosis [66], thus making it a potential therapeutic target. Li et al. [67] demonstrated that allicin exhibited an anti-myocardial fibrosis effect on MI rats by repressing the deposition of myocardial collagen fibres through downregulating expressions of collagen I, collagen III, TGF-β1, and Smad3 while upregulating expression of Smad7. Liu et al. [68] showed that allicin exerted a protective effect against cardiac dysfunction and cardiomyocyte apoptosis, while also inhibiting the progression of myocardial fibrosis in streptozotocin (STZ)-induced diabetic rats. The mechanism underlying its amelioration of myocardial fibrosis was associated with the inhibition of CTGF and TGF-β protein expression. In addition, the extent of fibrosis is closely related to inflammatory responses. NF-κB serves as a prototypical pro-inflammatory signalling pathway capable of activating the TGF-β pathway to facilitate myocardial fibrosis [69]. Kong et al. [19] found that allicin alleviated cardiac dysfunction and decreased myocardial fibrosis in STZ-induced diabetic rats by inhibiting the NF-κB signalling pathway (Table 5).

**Table 5:** The mechanisms of allicin in myocardial fibrosis

Research model	Model establishment	Intervention methods	Drug effects	Main molecular mechanisms	Citation
Wistar male rats with diabetic cardiomyopathy	Single injection of 65 g/kg streptozotocin intraperitoneally	40 mg/kg gavage for 4 weeks	Alleviating cardiac dysfunction, decreasing myocardium fibrosis	⬇ LVEDD, LVESD, NF-κB p65, p-NF-κB p65 ⬆ LVEF, FS, E/A	[19]
Wistar rats with MI	Ligating LAD	1.2, 1.8, and 3.6 mg/kg, intraperitoneal injection for 3 weeks	Alleviating myocardial fibrosis	⬇ Collagen I, collagen III, TGF-β, Smad3 ⬆ Smad7	[67]
Male Wistar rats with Diabetes mellitus	Single intraperitoneal injection of streptozotocin, 40 mg/kg after overnight fast	4, 8, and 16 mg/kg were given by intraperitoneal injection	Reducing myocardial damage, improving cardiac function	⬇ Blood glucose, Fas, TGF-β1, CTGF, Ca^2+^, LVEDP, − dp/dtmax ⬆ Bcl-2, LVSP, + dp/dtmax	[68]

*Bcl-2* b-cell lymphoma-2, *CTGF* connective tissue growth factor, *FAS* factor related apoptosis, *LVEDD* left ventricular end-diastolic dimension, *LVEDP* left ventricular end-diastolic pressure, *LVESD* left ventricular end-systolic internal diameter, *LVSP* left ventricular systolic pressure, *LVW/BW* left ventricle weight to body weight ratio, *LVPWd* left ventricular posterior wall diastolic thicknesses, *LVSP* left ventricular systolic pressure, *TGF-β* transforming growth factor-β, *± dp/dtmax* maximum rate of the left ventricular pressure rise and fall

### Arrhythmias

Arrhythmias often result from alterations in the electrophysiological properties of cardiomyocytes and their underlying ion channels [70]. Numerous studies have indicated that garlic and garlic extract can improve arrhythmias through modulation of ion channels [71]. Deng et al. [72] found that allicin inhibited the transient outward K^+^ current in a concentration-dependent manner and prolonged the action potential duration of human atrial myocytes, but it did not affect the ultrarapid delayed rectifier K^+^ current and the L-type Ca^2+^ current. Building upon this finding, Cao et al. [73] further demonstrated that allicin also exhibited an inhibitory effect on the transient outward potassium current (I_to_) in mouse ventricular myocytes. High-dose allicin (≥ 100 µmol/L) accelerated the voltage-dependent inactivation of I_to_ in mouse ventricular myocytes, which may be the potential mechanism by which allicin exerts its anti-arrhythmic effect. The Cav1.2 channel conducts the L-type calcium current (I_CaL_), which mediates excitation–contraction coupling and action potential duration, thereby playing a crucial role in cardiac electrophysiological activities [74]. Han et al. [75] indicated that allicin inhibited Cav1.2 channels by reducing the expression of channel proteins, providing a partial explanation for its inhibitory potential. Allicin also effectively inactivated the ΔKPQ-SCN5A mutant channel in congenital long QT syndrome type 3 (LQT3), thereby reducing the late sodium current of the ΔKPQ-SCN5A mutation [76]. Moreover, allicin decreased the ratio of late sodium current to peak current (INa,L/INa,P) by promoting Nav1.5 distribution on the cell membrane, resulting in therapeutic effects on LQT3. These findings indicate that allicin can ameliorate arrhythmias by regulating multiple ion channels (Table 6).

**Table 6:** The mechanisms of allicin in arrhythmias

Research model	Model establishment	Intervention methods	Drug effects	Main molecular mechanisms	Citation
Human atrial myocytes	–	30 μmol/l for 3 min (validation concentration)	Prolonging the action potential duration	⬇ I_to_	[72]
Ventricular cardiac myocytes from C57BL/6	–	10, 30, 100, and 300 μmol/l for 5 min at a rate of 2‑3 ml/min at room temperature	Anti-arrhythmia	⬇ I_to_	[73]
Primary cardiomyocytes from neonatal Sprague–Dawley rats	–	3, 10, 30 μmol/l for 48 h	Anti-arrhythmia	⬇ Cav1.2 channel protein trafficking	[75]
HEK293 with△KPQ-SCN5A mutations	The transferred △KPQ-SCN5A plasmid was transiently	30 μmol/l (validation concentration)	Increasing the channel steady-state and intermediate-state inactivation, reducing the window current	⬇ I_Na, L_, I_Na, L_/I_Na, P_ ⬆ Nav1.5 channel protein	[76]

Cav1.2: the l-type calcium; △KPQ-SCN5A: the cardiac Na^+^ channel; I_Na, L_: the late sodium current; I_Na, P_: persistent sodium current; I_to_: transient outward potassium current

### Cardiotoxicity

Cardiotoxicity is a severe side effect secondary to cardiac damage caused by chemotherapy drugs, ultimately leading to MI, myocardial fibrosis, and HF [77]. Therefore, there is an urgent need to explore natural extracts that can alleviate cardiotoxicity. In rat models of TZB-induced cardiotoxicity, allicin treatment not only reduced the levels of pro-inflammatory cytokines and myocardial enzymes, including TNFα, IL-1β, IL-6, cTnI, cTnT, and LDH [78], but also significantly attenuated cell apoptosis and ROS levels. These findings suggest that allicin possesses anti-inflammatory, anti-fibrotic, antioxidant, antihyperlipidemic, and anti-apoptotic properties that can potentially alleviate TZB-induced cardiotoxicity. Thubiani et al. [79] revealed that allicin-attenuated adriamycin (ADR) -induced myocardial injury by inhibiting oxidative stress and inflammation. Allicin pretreatment significantly suppressed the elevation of ADR-induced serum CK-MB and LDH levels, as well as diminished the expression of oxidative parameters such as TNF-α and TGF-β. Furthermore, allicin exhibited a protective role against DOX-induced cardiotoxicity by effectively alleviating cardiac oxidative damage, apoptosis, and inflammation [17]. These findings highlight the potential clinical application of allicin as a promising natural drug for anti-cardiotoxicity (Table 7).

**Table 7:** The mechanisms of allicin in cardiotoxicity

Research model	Model establishment	Intervention methods	Drug effects	Main molecular mechanisms	Citation
Male Swiss albino mice with cardiotoxicity	Intraperitoneal doxorubicin injection at a dose of 10 mg/kg at 7, 9, and 11th day	20 mg/kg, orally via gavage for 2 weeks	Mitigating cardiac oxidative damage, reducing apoptosis and inflammation	⬇ AST, LDH, CK, CK-MB MDA, NO, TNF-α, IL-1β, caspase-3, COX-2 ⬆ SOD, GSH, GPX, CAT	[17]
Female Wistar albino rats with cardiotoxicity	6 mg/kg/week trastuzumab by intraperitoneal injection	9 mg/kg, orally via gavage for 5 weeks	Anti-inflammation, anti-fibrotic, antioxidant, antihyperlipidemic, and anti-apoptotic properties	⬇ Inflammatory cells, collagen fibres, apoptotic cells, TC, LDL VLDL, TNF-α, IL-1β, IL-6, cTnT, cTnI, MB, LDH ⬆ HDL, SOD3, GPX1, CAT	[78]
Female Wistar rats with cardiotoxicity	15 mg/kg adriamycin intraperitoneally on day 8	20 mg/kg for 10 days	Adjusting ECG, anti-inflammation, reducing myocardial damage, suppressing oxidative stress	⬇ QT, QTC, QRS, T peak-Tend intervals, PR interval and duration, PR length, CK-MB, TNF-α, MDA ⬆ SOD, MDA, GSH	[79]

*CAT* catalase, *CK-MB* creatine kinase-MB, *COX-2* cyclo-oxygenase-2, *ECG* electrocardiography, *GPX1* glutathione peroxidase 1, *MDA* malondialdehyde, *SOD* superoxide dismutase

## Noval nanotechnology-based drug delivery strategies of allicin

Allicin has substantial potential for CVD therapy. Consequently, the majority of existing research has focused on elucidating allicin’s biological activity and potential health benefits rather than its pharmacokinetics. However, due to its lipophilic nature and poor water solubility, allicin exhibits limited dissolution and bioavailability when administered as a drug. Numerous studies have indicated that its half-life is less than 1 min, highlighting its inherent instability [80]. Moreover, being a stimulating ingredient, allicin may induce adverse reactions such as intolerance, allergy, and gastrointestinal disturbances [81]. These factors undoubtedly pose substantial challenges in effectively translating it into therapeutic modalities. Therefore, it is imperative to explore safer, more stable delivery systems with low toxicity and high loading capacity for allicin to enhance its applicability in CVD treatment.

**Fig. 2:**
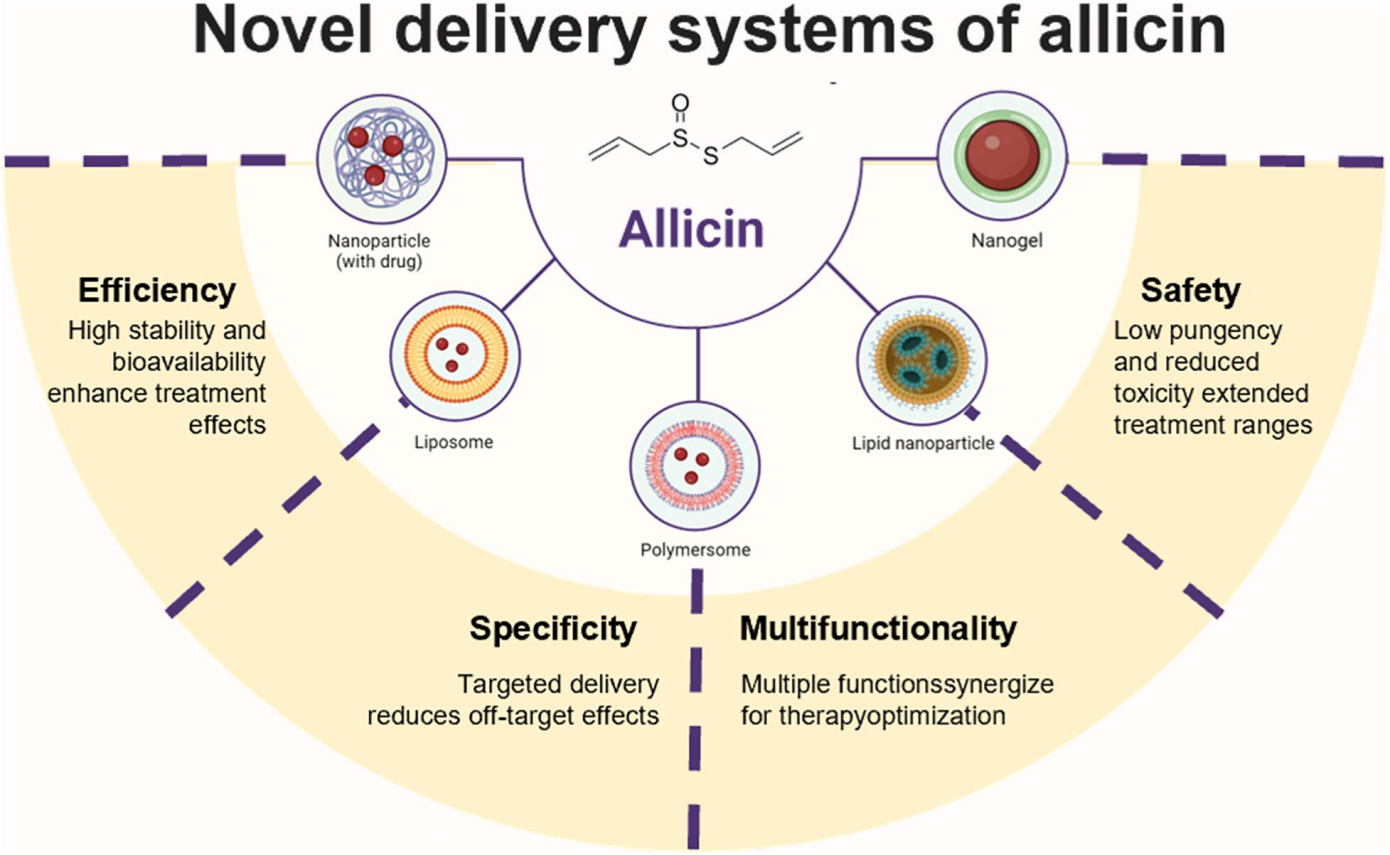
Novel nano-drug delivery systems for allicin enhance its efficiency, specificity, safety, and multifunctionality in clinical applications

In recent years, nano-biotechnology has garnered much greater attention as a burgeoning field with immense scope and application in drug delivery platforms. Advances in nanotechnology-based drug delivery systems show promise in addressing these challenges, potentially enhancing the solubility, stability, and bioavailability of allicin. Currently, various nanoformulations such as nanoemulsions [82], liposomes [83], hydrogels [84], gelatin nanoparticles [85], and other miscellaneous formulations [86] not only have the ability to encapsulate and protect allicin from degradation but also improve its solubility, stability, permeability, and retention at the target site [87] (Fig. 2).

### Nanoparticles

Nanoparticles (NPs) are solid particles ranging in size from 1 to 100 nm, possessing a substantial surface area that enables the encapsulation or adsorption of drugs and other active ingredients onto their substrate [88]. Consequently, NPs facilitate enhanced drug absorption and bioavailability. Due to their diminutive size, NPs possess not only the capability to traverse cell membranes or the blood–brain barrier for targeted drug delivery but also evade immune surveillance to prolong their retention times within biological tissues. Therefore, NPs have emerged as a promising drug delivery system in medicine, particularly for disease treatment and diagnosis [89]. Moreover, the advantages of nanoparticles as delivery systems lie in their ability to increase drug loading capacity, enhance stability, exert sustained control over release properties, and facilitate the transport of drugs across cell membranes or biological barriers through precise modulation of their morphology, surface chemistry pharmacokinetics, and release characteristics. A recent study discovered that the allicin-loaded polylactic acid–glycolic acid nanoparticles enhanced the encapsulation efficiency and drug-loading capacity of allicin, while also ensuring a consistent controlled-release rate of allicin in an acidic environment [87]. Hashemy et al. [90] developed polyethylene glycol and folic acid-modified chitosan-egg phospholipid nanoparticles loaded with allicin (AC-PLCF-NPs), which demonstrated potent inhibitory activity against Gram-negative bacterial strains and enhanced cytotoxicity against HT-29 cancer cells compared to free allicin. Chen et al. [86] developed a functionalized adipo-8 aptamer loaded with allicin to form a synergistic binary-drug delivery system for treating obesity. This study demonstrated that this nanoparticle-based system efficiently protected allicin from degradation, exhibiting significant potential in encapsulating, transporting, and releasing the molecular cargo into white adipose tissue. Despite the considerable potential of NPs in drug delivery applications loaded with allicin, it is imperative to consider their potential toxic effects. Various factors may influence the toxicity and biocompatibility of nanoparticles, necessitating thorough investigation before their application in biomedicine.

### Liposomes

Liposomes are spherical, double-layered vesicles formed by an amphiphilic phospholipid membrane, which contains an aqueous core and a hydrophobic surface. Due to the excellent cell affinity of the lipid-like bilayer membrane, liposomes exhibit remarkable biodegradation, biocompatibility, and sustained release characteristics [91]. Simultaneously, they effectively shield drugs from external environmental factors such as enzymatic degradation, pH changes, chemical hydrolysis, and oxidative degradation thereby enhancing drug stability. Additionally, liposomes can encapsulate, deliver, and release various water-soluble materials and lipid-soluble and amphiphilic substances [92]. These advantages establish liposomes as one of the most commonly utilized nano drug delivery systems. Cristian et al. [83] developed a liposome nanodrug delivery system, demonstrating that liposomes serve as a suitable encapsulation system for stabilizing garlic active compounds and enhancing the stability of allicin. The application of liposomes, however, still encounters several challenges such as limited drug loading capacity, inadequate long-term stability, and high costs associated with large-scale industrial production [93].

### Hydrogels

Hydrogel is a type of polymer characterized by its three-dimensional network structure formed through the cross-linking of the polymer backbone and hydrophilic functional groups via covalent bonds, ionic bonds, hydrogen bonds, or physical entanglement [94]. The hydrogel-based drug delivery systems have emerged as a promising approach for targeted and sustained drug release due to their exceptional biocompatibility, adjustable physicochemical properties, and ability to encapsulate both hydrophilic and hydrophobic drugs [95]. With further research, hydrogel shows promising potential in myocardial repair due to its similar three-dimensional structure to the extracellular matrix, excellent biocompatibility and mechanical properties, as well as its ability to provide a physiological environment akin to that of native myocardium [96]. A recent study indicated that the application of a smart hydrogel coating loaded with allicin can have the potential to facilitate endothelial cell regeneration, suppress smooth muscle cell proliferation, and mitigate the inflammatory response surrounding blood vessels to promote neovascularization in vivo [84]. This hydrogel system had excellent biocompatibility and could regulate the atherosclerotic microenvironment and prevent in-stent restenosis by continuously releasing allicin. Although significant advancements have been made in the field of hydrogels for cardiac tissue engineering, several critical challenges remain to be addressed before their safe implementation in clinical practice. For instance, further investigation is required regarding the selection of appropriate materials for cardiac repair, optimal dosage and timing of injection, and efficient distribution strategies [97].

### Nanoemulsions

Nanoemulsions (NEs) is a stable, transparent, and low-viscosity dispersion system composed of the oil phase, water phase, surfactant, and cosurfactant in appropriate proportions. It has been found that NEs, as a novel nano-drug delivery system, have the characteristics of enhancing the solubility, stability, and bioavailability of poorly soluble drugs due to their small and uniform particle size, and thus have the functions of targeting and sustained release drug delivery [98]. Due to these advantages, NEs are rapidly emerging as a versatile platform for drug delivery and biomedical applications. Ke et al. [82] discovered that the in vitro drug release kinetics of allicin from the NEs drug delivery system exhibited a delayed release under various biological pH conditions. Moreover, allicin demonstrated prolonged and sustained drug release, particularly in an inflammatory environment. However, NEs also present certain limitations, such as the potential risk of surfactant-induced toxicity and inadequate long-term stability, thereby rendering it unsuitable for serving as a carrier for sustained-release drugs over extended periods [99].

In conclusion, drug delivery strategies based on nanotechnology offer the potential to revolutionize the way allicin is administered and targeted within the body. Nevertheless, while allicin-loaded nanoformulations have demonstrated potential in treating various diseases, the cardioprotective mechanisms of allicin-based nanoformulations remain poorly understood and require further research. In the future, preclinical assessment of allicin-loaded nanoformulations in animal models could be a viable research approach to comprehend allicin’s stability and bioavailability fully. Additionally, conducting a comparative analysis of various drug delivery methods and designing targeted delivery systems can offer additional insights to enhance allicin’s therapeutic potential.

## Limitations of clinical application

Finding feasible and effective natural pharmaceutical ingredients for the treatment of CVD has always been one of the most challenging issues in medical research. Due to the unsatisfactory efficacy of most existing drugs targeting various CVD, natural ingredients like allicin have emerged as a promising treatment option for CVD patients. However, the clinical application of allicin still has certain limitations. Firstly, due to the unstable chemical properties and unpredictable bioavailability of allicin, further research is needed on purification processes and preparation methods to improve its stability and pharmacological activity. Recent advances in formulation strategies and nanotechnology-based drug delivery systems show promise in addressing these challenges, potentially improving allicin’s solubility, stability, and bioavailability [100, 101]. Meanwhile, the targeting technology of nanoscale materials enabled the delivery of active ingredients to the desired action site in sufficient concentrations, enhancing their ability to get absorbed into cells. These form the rationale and potential approaches to developing innovative delivery strategies to boost the bioavailability and stability of allicin [102].

Secondly, while various researchers have targeted several signalling pathways to investigate the mechanism behind the cardiovascular protective efficacy of allicin, additional research is still required to comprehend these molecular targets of allicin inside diverse CVD fully. Furthermore, despite promising preclinical research on the cardioprotective potential of garlic components, the paucity of well-studied, potent clinical studies and thorough safety assessments increases the desire for additional clinical research [103]. Therefore, to ensure the safety and efficacy of allicin in preventing and treating CVD and to better understand its effects, it is imperative to conduct large-scale randomized clinical trials and further basic research.

## Conclusions

CVD severely threatens human health. These diseases may be alleviated with ingredients derived from natural medicines. Allicin is a natural bioactive compound with cardioprotective effects, especially anti-inflammatory, anti-apoptotic, and anti-oxidative stress effects through multi-targets and multi-mechanisms. Although it has shown promising potential in the treatment of numerous CVDs such as atherosclerosis, myocardial infarction, hypertension, heart failure, arrhythmias, cardiac hypertrophy, myocardial fibrosis, and cardiotoxicity, further studies and trials are required to validate the beneficial treatment of allicin in CVD which have not been exhaustively examined. More importantly, the problems of instability, poor bioavailability, and irritating odor are supposed to be resolved in future experimental studies. Several novel delivery systems for allicin have been developed, ensuring high stability, loading capacity, and bioavailability. Future research should prioritize the development of delivery systems with high loading capacities for allicin while maintaining a precisely targeted therapy for CVD. Overall, allicin is a prospective choice for treating CVD and will likely be used to prevent and manage CVD in the future.

## Data Availability

Not applicable.

## Abbreviations

ACEIs: Angiotensin-converting enzyme inhibitors
ApoE: Apolipoprotein E-deficient
Ang II: Angiotensin II
AS: Atherosclerosis
AT2R: Angiotensin II receptor type 2
BNP: Brain natriuretic peptide
CAT: Catalase
CCBs: Calcium channel blockers
CH: Cardiac hypertrophy
cIMT: Carotid intima-media thickness
DAMPs: Damage-associated molecular patterns
DDS: Diallyl disulfide
DOX: Doxorubicin
DTS: Diallyl trisulfide
eNOS: Endothelial nitric oxide synthase
GSH-Px: Glutathione peroxidase
H_2_O_2_: Hydrogen peroxide
H_2_S: Hydrogen sulfide
Hcy: Homocysteine
HF: Heart failure
HR: Hypoxia-reoxygenation
IL-6: Interleukin-6
JNK: Jun N-terminal kinase
LDL-C: Low-density lipoprotein cholesterol
LDLR: Low-density lipoprotein receptor
LXRα: Liver X receptor alpha
MDA: Malondialdehyde
mTOR: Mammalian target of rapamycin
MI: Myocardial infarction
MI/R: Myocardial ischemia–reperfusion
MTX: Methotrexate
NF-κB: Nuclear factor kappa B
NLRP3: NOD-like receptor pyrin domain-containing protein 3
NO-sGC-cGMP: NO-soluble guanylate cyclase-cyclic guanosine monophosphate
PCSK9: Proprotein convertase subtilisin/kexin type 9
PGI2-AC-cAMP: Prostacyclin-adenylyl cyclase-cyclic adenosine monophosphate
PPARγ: Peroxisome proliferator-activated receptor γ
Prx: Peroxidase
ROS: Reactive oxygen speciesc
SAC: S-allyl cysteine
SOD: Superoxide dismutase
SREBP2: Sterol regulatory element binding proteins 2
TC: Total cholesterol
TG: Triglycerides
TGF-β: Transforming growth factor-β
TLR4: Toll-like receptor 4
TMAO: Trimethylamine-*N*-oxide
TNF-α: Tumor necrosis factor-alpha
